# CT-Based Collision Prediction Software for External-Beam Radiation Therapy

**DOI:** 10.3389/fonc.2021.617007

**Published:** 2021-03-11

**Authors:** Yu-Jen Wang, Jia-Sheng Yao, Feipei Lai, Jason Chia-Hsien Cheng

**Affiliations:** ^1^ Graduate Institute of Biomedical Electronics and Bioinformatics, National Taiwan University, Taipei, Taiwan; ^2^ Department of Radiation Oncology, Fu Jen Catholic University Hospital, New Taipei City, Taiwan; ^3^ School of Medicine, College of Medicine, Fu Jen Catholic University, New Taipei City, Taiwan; ^4^ Department of Computer Science and Information Engineering, National Taiwan University, Taipei, Taiwan; ^5^ Division of Radiation Oncology, Departments of Oncology, National Taiwan University Hospital, Taipei, Taiwan; ^6^ Graduate Institutes of Oncology, Taipei, Taiwan; ^7^ Clinical Medicine, National Taiwan University College of Medicine, Taipei, Taiwan

**Keywords:** radiotherapy, collision, noncoplanar, beam angle, software

## Abstract

**Purpose:**

Beam angle optimization is a critical issue for modern radiotherapy (RT) and is a challenging task, especially for large body sizes and noncoplanar designs. Noncoplanar RT techniques may have dosimetric advantages but increase the risk of mechanical collision. We propose a software solution to accurately predict colliding/noncolliding configurations for coplanar and noncoplanar beams.

**Materials and Methods:**

Individualized software models for two different linear accelerators were built to simulate noncolliding gantry orientations for phantom/patient subjects. The sizes and shapes of the accelerators were delineated based on their manuals and on-site measurements. The external surfaces of the subjects were automatically contoured based on computed tomography (CT) simulations. An Alderson Radiation Therapy phantom was used to predict the accuracy of spatial collision prediction by the software. A gantry collision problem encountered by one patient during initial setup was also used to test the validity of the software. Results: In the comparison between the software estimates and on-site measurements, the noncoplanar collision angles were all predicted within a 5-degree difference in gantry position. The confusion matrix was calculated for each of the two empty accelerator models, and the accuracies were 98.7% and 97.3%. The true positive rates were 97.7% and 96.9%, while the true negative rates were 99.8% and 97.9%, respectively. For the phantom study, the collision angles were predicted within a 5-degree difference. The software successfully predicted the collision problem encountered by the breast cancer patient in the initial setup position and generated shifted coordinates that were validated to correspond to a noncolliding geometry.

**Conclusion:**

The developed software effectively and accurately predicted collisions for accelerator-only, phantom, and patient setups. This software may help prevent collisions and expand the range of spatially applicable beam angles.

## Introduction

Collision prevention for patient safety is a key issue in radiation therapy (RT) (1). The spatial optimization of beam angles remains essential and critical for RT; however, challenges of increased uncertainty can arise with large-size patients or noncoplanar beams, leading to potential gantry collisions. Noncoplanar RT techniques, which potentially have dosimetric advantages by virtue of the additional degrees of freedom, have been used in several clinical situations, including the treatment of lung cancer and liver cancer (2, 3). However, the increased risk of gantry collision demands special attention and prohibits frequent use. Obstacles remain with regard to the necessary replanning work and the delay in treatment when a problem of unexpected gantry collision is encountered. The conservative use of noncoplanar beams or pretreatment dry runs compromises the broad application of such techniques. Hence, the ability to select noncoplanar beam angles that will not result in gantry collisions mainly depends on individual skill and experience. A few preliminary methods have been proposed to prevent collisions (2, 4–10), such as a chart of couch-gantry combinations (7), prediction software for collision avoidance (8, 10), and the use of supplemental cameras (5, 11) or a 3-dimensional scanner (4). Graphical simulations (12, 13) or experimental reference measurements (14) also offered some possible approaches to try to solve above problem. Unfortunately, no user-friendly, patient- and equipment-specific method has yet been presented that can conveniently predict collisions. Our goal is to establish a software platform that can integrate patient- and accelerator-specific information into the estimation of the noncolliding space for coplanar/noncoplanar beam selection before the planning process in order to improve patient safety and prevent hardware damage.

## Materials and Methods

### Geometrical Linear Accelerator Models

Geometrical models of two accelerators were built for simulations based on polygonal geometry. A model with a cantilever, with the accelerator head anchored at one end and a vertical support, and a rotatable couch was built for each system. The machine dimensions were set in accordance with the manuals and on-site measurements. Linear accelerators from two different vendors, the Elekta Synergy^®^ (Elekta, Crawley, UK) and the Varian TrueBeam™ STx (Varian Medical Systems, Palo Alto, CA, USA), were used. The models were established in Unity™ 3D (Unity Technologies, San Francisco, CA, USA).

### Automatic Computed Tomography (CT) Contouring and Image Exporting

The simulation CT images were subjected to image segmentation, image intensity transformation, and region of interest (ROI) processing, and the contours were exported as Digital Imaging and Communications in Medicine (DICOM) files. For comprehensive collision prediction, the contoured subjects included the body surface, auxiliary equipment such as a shell or a vacuum bag, and other accessories above the CT couch plate. A user interface (UI) was designed to help users choose their own intensity transformation specifications. We applied the grayscale morphological closing technique from the field of computer vision to smooth the images. The functions of dilation, erosion, and contour finding for grayscale images were implemented using the Open Source Computer Vision (OpenCV) library. The isocenters of the CT images were defined manually. Finally, the DICOM images were exported and saved in the secured space.

### Collider Models

Each DICOM file was exported with three contour sequences including the isocenter, the CT plate height, and a collider. A collider is any object which can collide with the gantry, either the patient body or any auxiliary equipment. Each contour is retrieved from the CT image in a 2-dimensional space. Each contour on the single CT slice is a set of consecutive points located on an XY plane. In order to construct the 3-dimensional (3D) structure from 2-dimensional (2D) images, we implemented an algorithm to incorporate the Z-axis coordinate into the 2D image stacks with contour interpolation. Linear interpolation was used between the two corresponding contours on the closest adjacent slices to convert a set of 2D contours into a 3D mesh (15). A 3D mesh in Unity™ 3D therefore is created based on the patient’s external contour and consists of a set of vertices and a set of triangles. Triangulation is applied to split the polygon into triangles.

### Collision Detection

We used a mesh collider function built in Unity™ 3D to perform collision detection (16). In our study, we used the trigger for a mesh to detect collision events. We did not use the collider function for a rigid body because Unity™ 3D does not allow two rigid bodies to overlap. Our work was designed to simulate all the possible gantry and couch combinations including configurations involving collisions. Three types of triggers within Unity were used: [OnTriggerEnter], [OnTriggerStay], and [OnTriggerExit]. When an object collides with another object, Unity calls [OnTriggerEnter]. [OnTriggerExit] is called when the colliding event ends. In between [OnTriggerEnter] and [OnTriggerExit] is called [OnTriggerStay].

### Model Validations for an Empty Couch, A Phantom, and A Patient

Collision was manually assessed for 38 couch- and gantry-angle configurations of the two different linear accelerators based on approximately 400 measurements from different couch coordinates. An Alderson Radiation Therapy (ART) phantom immobilized with a vacuum bag was used to test the model collision estimation. The couch heights were chosen to be at the lowest reachable position, 10 cm below the isocenter, and 20 cm below the isocenter; these positions span the heights at which most treatments are performed. The couch angles ranged from 0 to 90 degrees counterclockwise and 270 degrees clockwise. The gantry angles were tested up to the points at which couch-gantry or gantry-phantom collisions were encountered within the 3-cm safety zone or when the “Collision” alarm was displayed. The couch rotation angles were measured in increments of 10 degrees from 0 to 350 degrees. For the Varian TrueBeam™ STx, the official “LaserGuard Protection Zones” rules were applied for reference. DICOM images of one breast cancer patient, who underwent postlumpectomy adjuvant radiotherapy in the prone position and encountered a real gantry-patient collision problem on the first treatment day, were used to verify the validity of our models. The frequency of inaccurate predictions was counted by difference in every specific gantry-couch combination between prediction and on-site measurement. False negative (FN) was defined as no collision was estimated but a collision was warned in the on-site measurement. The false negative rate and false positive (FP) rate could be adjusted by expanding or shirking the safety zone.

$$\text{Accuracy}=\frac{\text{True}\;\text{positive}\;(\text{TP})+\text{True}\;\text{negative}\;(\text{TN})}{\text{TP}+\text{TN}\;+\text{FP}+\text{FN}}$$

$$\text{TPR}=\frac{\text{TP}}{\text{TP}+\text{FN}}\text{TNR}=\frac{\text{TN}}{\text{TN}+\text{FP}}$$

**Figure 1:**
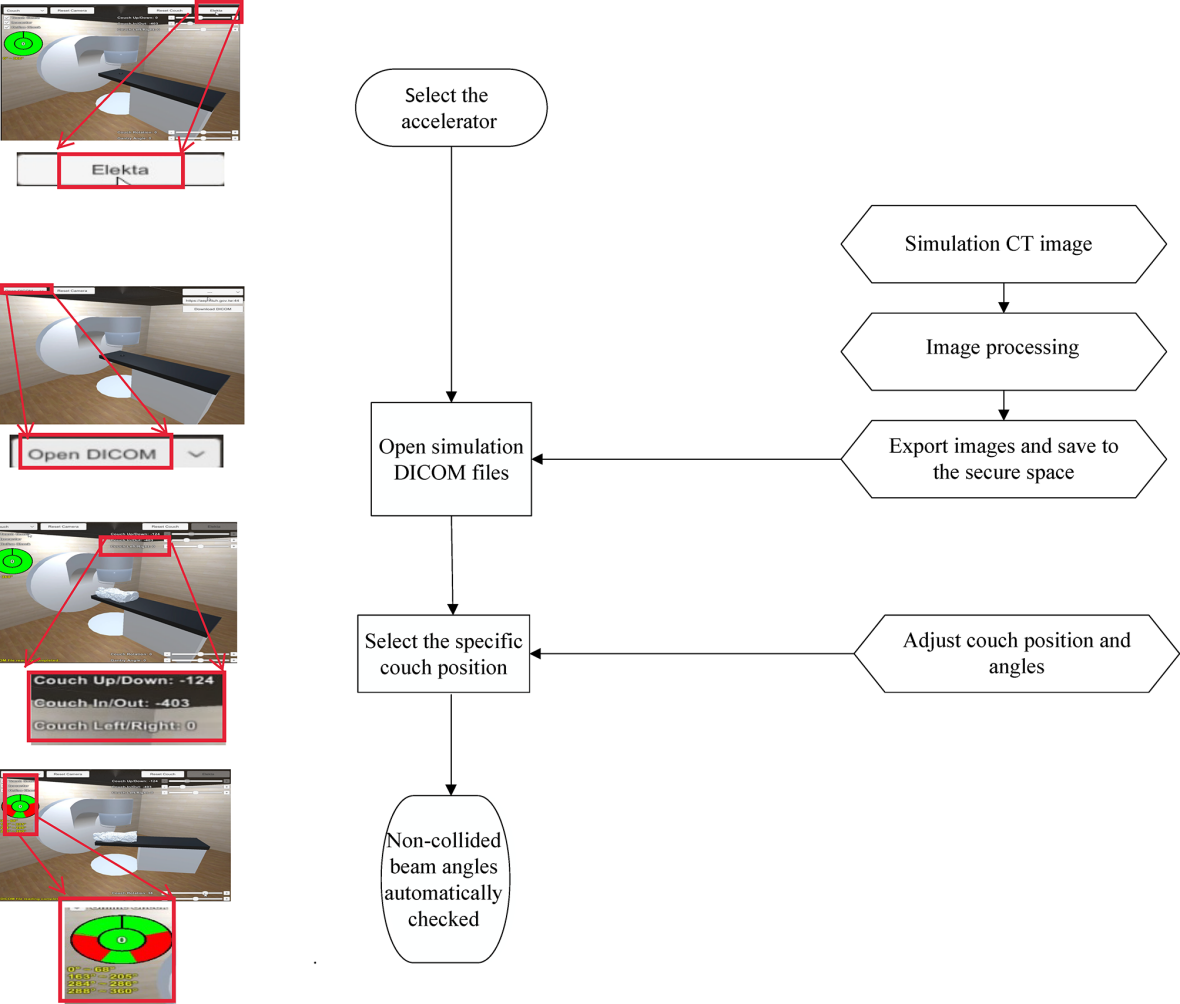
The executable actions with the Human Machine Interface (HMI) interface of our software.

The workflow of the software was shown as **Figure 1**. The study was approved by the Institutional Review Board (201811050RINB).

## Results

### Linear Accelerator Delineation

**Figure 2:**
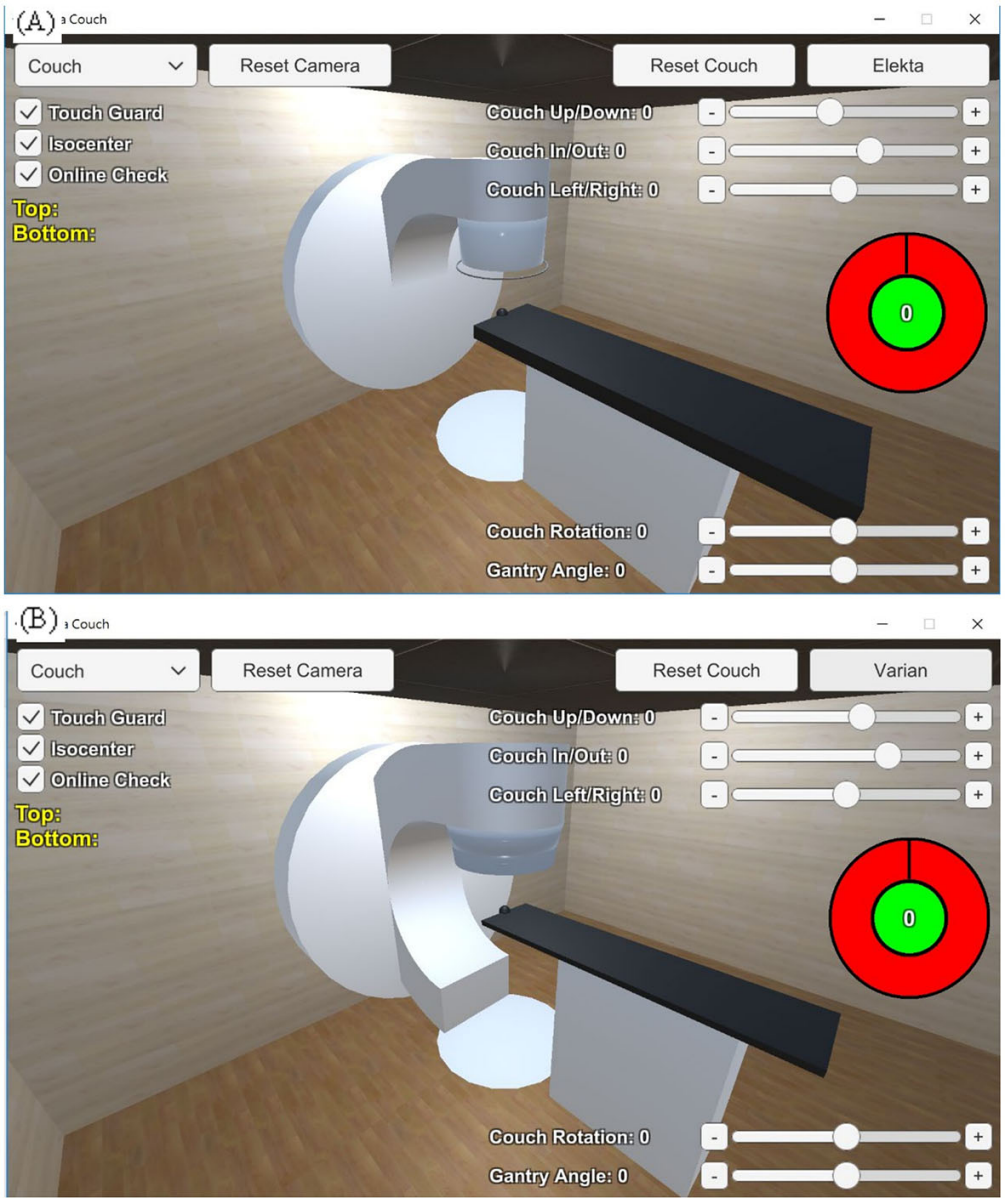
Linear accelerator models of **(A)** the Elekta Synergy^®^ and **(B)** the Varian TrueBeam™ STx delineated with different polygonal pieces using Unity™ 3D.

The linear accelerator models were divided into different parts for size and shape delineation, including the accelerator, the gantry, the touch guard mounted on the gantry, the couch plate, and the patient support. The couch rotator and base were modeled as a polygonal piece and a cuboid, respectively. The couch plate was a cuboid, with a black sphere representing the isocenter. Three parts formed the gantry from each vendor, namely, the gantry gun, gantry arm, and gantry wall. The gantry was able to rotate with a 360-degree range. The couch plate could be freely adjusted in the up-down, right-left, and in-out directions, and the couch rotator could be freely adjusted for angles. The on/off switch for the collision detector was designed as shown in **Figure 2**.

### Surface Contours And Image Transfer

The external surface of the phantom or patient was first automatically contoured based on the simulation CT images. A platform application was established showing the current slice number, the total number of slices, the intensity transform values, the plate height, and the position of the isocenter. The user could adjust the intensity transform values to achieve CT contouring and apply computer vision processing to obtain outline images. The images were stored in DICOM format and could be uploaded to the cloud or downloaded from the cloud to the software.

### Software Validation for Collisions With An Empty Couch

**Figure 3:**
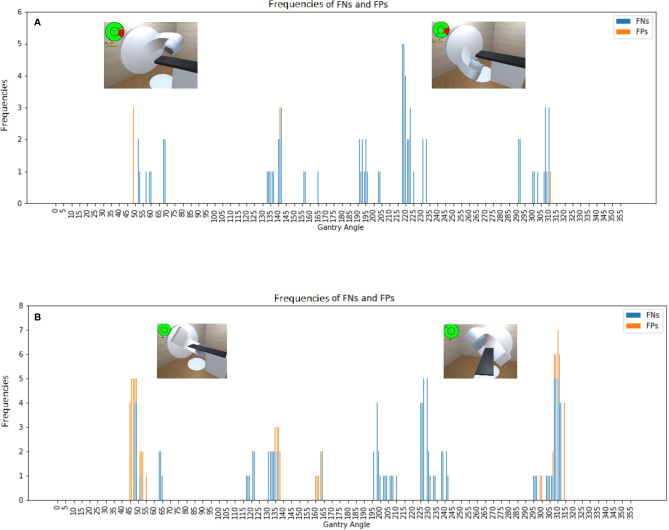
Frequencies of false negative predictions (FNs) and false positive predictions (FPs) for an empty couch for the **(A)** Synergy and **(B)** TrueBeam accelerators, with the representative collision conditions at gantry angles of 55°, 130°, 220°, and 310° illustrated.

**Figure 3** shows the confusion matrices based on software predictions and on-site measurements at 19 couch angles (every 10 degrees of rotation on either side) for the two linear accelerators without either a phantom or a patient on the couch. A total of 13,680 measurements were performed to verify the predicted collisions against the true collisions. The frequency of inaccurate predictions for the TrueBeam accelerator reached a maximum at a couch angle of 330 degrees and gantry angles of 220 degrees and 310 degrees, which were the positions with the smallest distance between couch and gantry. Similarly, for the Synergy accelerator, the worst predictions were found for a couch angle of 330 degrees and gantry angles of 310 degrees and 220 degrees. Among all the assessable couch and gantry angles, the software models achieved accuracies of 98.7% and 97.3% for the Synergy and TrueBeam accelerators, respectively. The true positive rates (TPRs) for the Synergy and TrueBeam accelerators were 97.8% and 96.9%, respectively, and the true negative rates (TNRs) were 99.8% and 97.9%, respectively.

### Software Validation for Collisions With A Phantom

**Figure 4:**
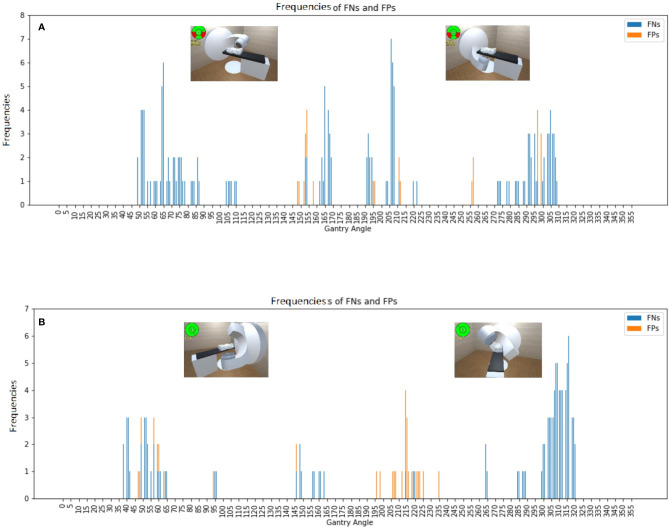
Frequencies of false negative predictions (FNs) and false positive predictions (FPs) with an Alderson Radiation Therapy phantom on the couch for the **(A)** Synergy and **(B)** TrueBeam accelerators, with the representative collision conditions at gantry angles of 65°, 150°, 210°, and 310° illustrated.

**Figure 5:**
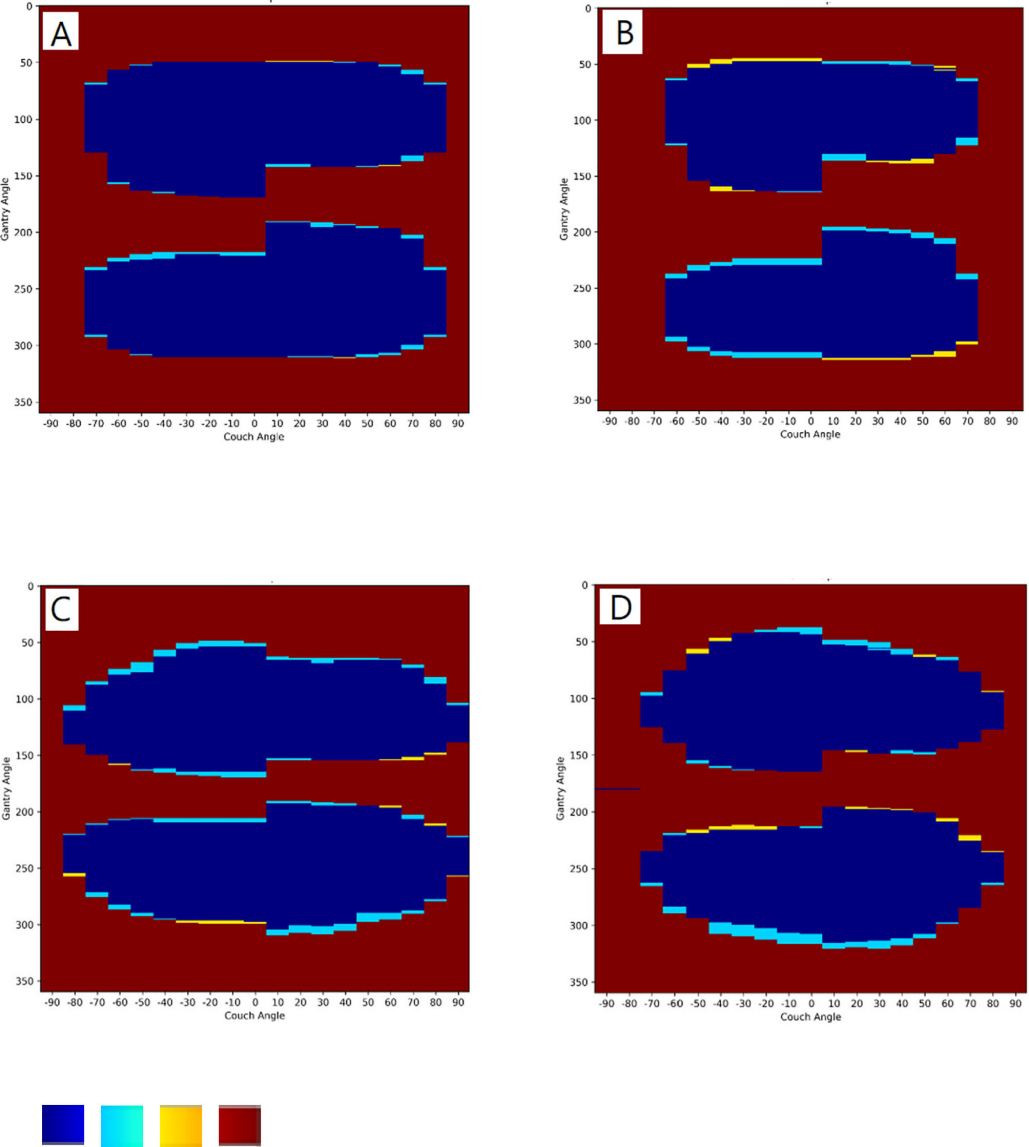
Collision predictions in matrix with an empty couch for **(A)** Elekta Synergy and **(B)** Varian TrueBeam accelerators, and with an Alderson Radiation Therapy phantom on the couch for **(C)** Elekta Synergy and **(D)** Varian TrueBeam accelerators. Color in blue, true positive (TP) collision; light blue, false positive (FP) collision; yellow, false negative (FN) collision; brown: true negative (TN) collision.

The software was also validated for collisions with an ART phantom immobilized with a vacuum bag on the couch for both accelerators (**Figure 4**). The software models achieved accuracies of 96.8% and 97.3% for the Synergy and TrueBeam accelerators, respectively. The TPRs for the Synergy and TrueBeam accelerators were 95.0% and 96.5%, respectively, and the TNRs were 99.2% and 98.6%, respectively. Collision predictions in matrix with an empty couch for (A) Elekta Synergy and (B) Varian TrueBeam accelerators, and with an Alderson Radiation Therapy phantom on the couch for (C) Elekta Synergy and (D) Varian TrueBeam accelerators were shown in **Figure 5**. Above operating process was described in our supplementary video file (**Supplementary Video 1**).

### Software Validation for A Breast Cancer Patient With A Collision Problem

**Figure 6:**
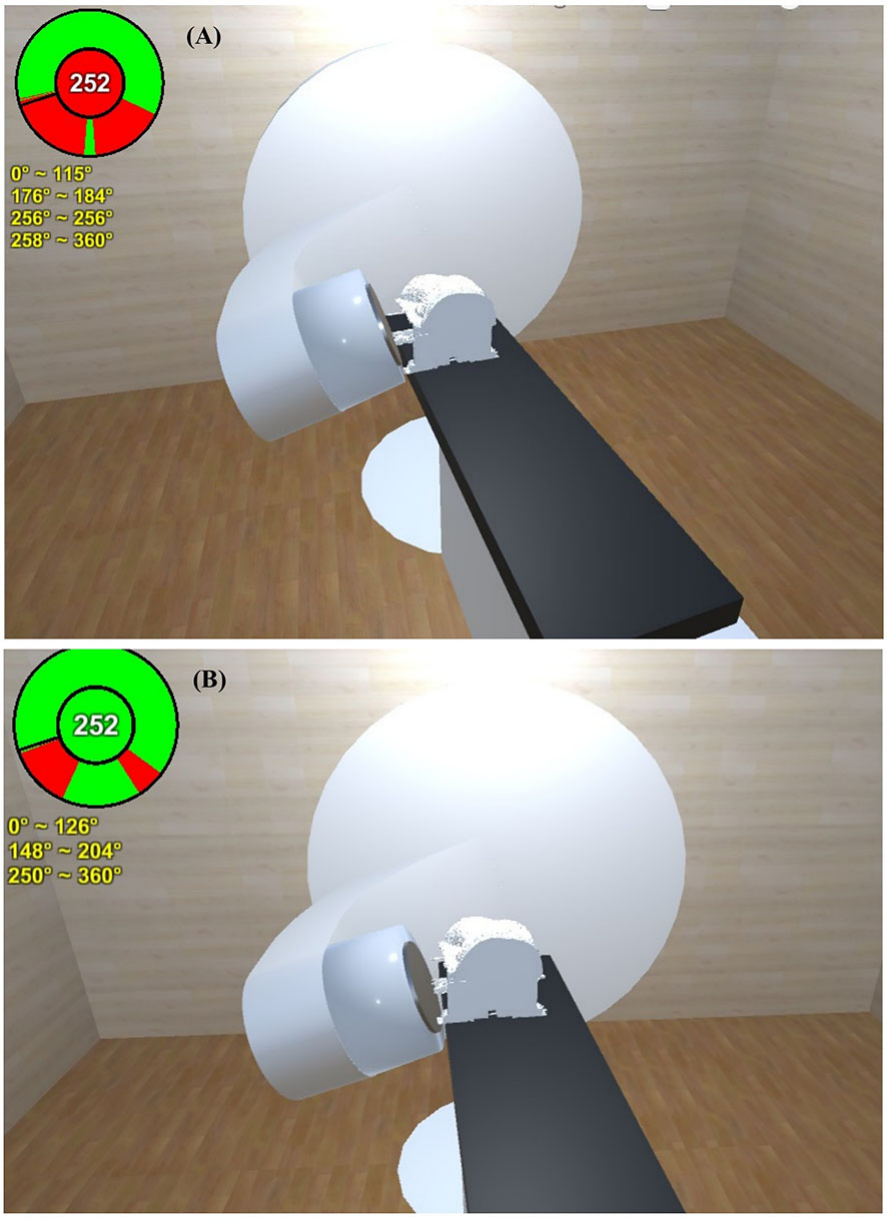
A collision between the gantry and couch of a representative breast cancer patient was encountered in the high setup position with the prone base. **(A)** This collision was accurately predicted by the software. **(B)** The downward shift of the isocenter necessary to avoid collision was also effectively estimated by the software.

Data from a breast cancer patient who encountered a gantry collision problem in the initial setup position in the Bionix prone breast system (Bionix, Toledo, OH, USA) were selected to validate the predictive power of the software. The external surface of the patient and the prone base were automatically contoured and exported to the software. On the first day of treatment, a collision between the gantry and couch was encountered in the high setup position with the prone base. This collision was accurately predicted by the software (**Figure 6A**), and the downward shift of the isocenter necessary to avoid collision was also effectively estimated by the software (**Figure 6B**).

## Discussion

We designed an automatic, user-friendly, and vendor-independent software tool that can produce accurate predictions of mechanical collision. The software performance is satisfactory, with all gantry-angle discrepancies being less than 5 degrees. With this convenient and effective tool, radiation oncologists and physicists will be able to adapt their procedures to complex body shapes and more efficiently adopt noncoplanar beam designs during the treatment planning process.

Several methods have been proposed in the attempt to solve the problem of possible collisions in clinical settings. Nguyen et al. (11) used supplemental live-view cameras to reduce blind spots. Cardan et al. (5) adapted a set of consumer depth cameras to create a polygon mesh of each object and achieved a high TNR for predictions. In addition, 4π radiotherapy is a useful therapeutic approach for ensuring target-dose conformity while sparing organs at risk (OARs), in which collision avoidance can be achieved through the use of 3D cameras (2). However, all of these solutions require additional devices. To the best of our knowledge, our software tool is the first to be able to simply use simulation CT images to predict mechanical collisions for various accelerators.

Our 3D software platform is based on Unity™ 3D (not Windows) mainly because Unity is well developed for real-time 3D projects for games, animation, film, architecture, engineering, and construction. It can be easily used for rendering 3D images and with the function to help collision detection conventionally used in computer gaming.

The performance indicators of our software, including the accuracy, TPR, and TNR, were all higher than 95% for two different accelerators and were comparable to those reported by Cardan et al. (5). Cardan et al. built a model with an average accuracy and TNR of 97.3% and 96.9%, respectively. Mann et al. achieved 94.2% accuracy (17). TNR in our study was less than their previously reports. However, the unavailability of the size and shape details of some small components of both accelerators made it difficult to achieve higher predictive power for collisions using our virtual models.

The F. Hueso-González et al. (18) offered an similar innovation with both photon and proton machine simulation data by beautiful machine stereolithography (STL) data with treatment planning system (TPS) system validation. However, our research with the strength as follows: 1. With auxiliary equipment such as a shell or a vacuum bag 2. Getting patient body contour information from simulation CT directly. 3. Validation by on-site two different brands linear accelerators measurements and a true patient scenario. The comparison between two studies listed in **Tables 1** and **2**.

**Table 1:** The similarity and difference between our study and the work by Hueso-González et al.

	Similarity	Difference
Our study	is a software for collision avoidance with the characteristics of easily adaptable, 3-dimensional (3D) visualization, and a tool for the treatment planners to choose beams wisely Offers the choice between automatic or visual collision detection Provides a realistic 3D visualization of treatment machine and patient Is patient-specific	Acquires images of patient and auxiliary device/equipment from the simulation CT scan It is not embedded in treatment planning system (TPS). Needs each of the treatment elements scanned in CT simulation process
Hueso-González et al. study		Acquires images of patient and auxiliary device/equipment from 3D camera and smartphone scan Is an internal software embedded in the TPS Allows the independent movement of each treatment room element, with real-time feedback

**Table 2:** Our proposal versus Hueso-González et al.: a comparative analysis.

	Strength or advantage	Weakness or disadvantage
Our Study	Calculates the auxiliary device/equipment such as an immobilization shell or vacuum bag Acquires patient body contour directly from simulation CT On-site measurement and validation with two different-vendor linear accelerators and a true patient scenario Expandable to add the other auxiliary equipment such as the vital sign monitor to the prediction model No need for a TPS for downloading images from the secured space	Needs the secured space to transfer patient data Is based on user’s manual and on-site measurement for concise polygonal geometry information of linear accelerators
Hueso-González et al. study	Capable of dealing with both photon and proton machines Beautiful 3D machine stereolithography (STL) data Rapid validation with treatment planning system	No on-site measurement of true accelerator or real patient Needs careful 3D surface scan to obtain surface information

The determined value of the confusion matrix was made based on the comparison between the model prediction and on-site measurement. The model built-up was not perfect due to doing polygonal geometry simulation for some machine parts with technical difficulty and some uncertainties from real world machine assembly processing. The accuracy may improve if a finer mesh grid was used with much more computational complexity (19, 20). Therefore, the touch guard and safe zone were offered by the two linear accelerators respectively to ensure the patient safety. In our model, we set-up 5-degree difference tolerance and adjust our polygonal geometry simulation. False negative rate in our study with empty couch was 2.2% and 3.1% respectively, and it could be close to almost zero if we sacrificed 5 predictive gantry degrees based on our validation results or expand the safety zone to reach the 99.3% TNR of TrueBeam accelerators but sacrifice the TPR down to 96.0%. It deserves attention that the indeterminations may be associated with different phases of simulation for our software. The input uncertainties include the precise geometry modeling of the accelerator before simulation, the relative position of auxiliary device to the patient in simulation, and body shape change from weight gain/loss after simulation (21).

The currently designed software has a few limitations. Some clinical collision scenarios may arise due to auxiliary equipment, such as the vital sign monitor, the breath-control device, or the image-acquisition guidance system. These devices may not be shown on simulation CT images and thus will not be considered by the software for collision estimation. We are making ongoing efforts to improve the functionality of the software to enable the incorporation of other in-room systems after CT simulation. Finer mesh grid may need to be established if the couch motion scale of the linear accelerator could be less than one degree. Notably, our system performs only collision prediction; it is not capable of spatially optimized beam selection for organ-sparing dosimetric purposes. In future work, we intend to integrate the target/OAR contours into the calculation process of the software with overlap metrics to generate noncolliding beam configurations for the spatial separation of OARs from targets and enhance advantageous dosimetric plan optimization.

In summary, our software is a convenient, vendor-independent, and high-performance tool for predicting gantry-couch and gantry-patient collisions using simulation CT images. It may help practitioners select noncolliding beam configurations and prevent unexpected mechanical collisions and treatment delays.

## Data Availability Statement

The original contributions presented in the study are included in the article/**Supplementary Material**. Further inquiries can be directed to the corresponding author.

## Ethics Statement

The studies involving human participants were reviewed and approved by the National Taiwan University Hospital Research Ethics Committee. Written informed consent for participation was not required for this study in accordance with the national legislation and the institutional requirements.

## Author Contributions

Y-JW was responsible for drafting the article and for analysis and interpretation of the data. J-SY was involved in model building, data acquisition, and analysis of the data. J-SY, FL, and JC were involved in the interpretation of the data. All authors contributed to the article and approved the submitted version.

## Conflict of Interest

The authors declare that the research was conducted in the absence of any commercial or financial relationships that could be construed as a potential conflict of interest.

## Supplementary Material

Supplementary Video 1 Operating process including step 1. select the accelerator, step 2. open simulation DICOM files, step 3. select the specific couch position, and step 4. non-collided beam angles automatically checked was described.
